# Use of extruded eggshell as a calcium source substituting limestone or oyster shell in the diet of laying hens

**DOI:** 10.1002/vms3.544

**Published:** 2021-05-25

**Authors:** Mohammad Aminul Islam, Masahide Nishibori

**Affiliations:** ^1^ Department of Dairy and Poultry Science Bangabandhu Sheikh Mujibur Rahman Agricultural University Gazipur Bangladesh; ^2^ Lab of Animal Genetics Graduate School of Integrated Sciences for Life Hiroshima University Higashi‐Hiroshima Japan

**Keywords:** Calcium sources, egg production, egg quality, laying hen, net profit

## Abstract

The nutritional values of limestone, oyster shell and extruded eggshells were evaluated using different methods. In total, 120 ready‐to‐lay pullets, 18‐week‐old ISA Brown were distributed into six dietary groups, namely D_1_ (4% limestone), D_2_ (8% limestone), D_3_ (4% oyster shell), D_4_ (8% oyster shell), D_5_ (4% eggshell) and D_6_ (8% eggshell), to assess the effect of calcium sources on egg production, egg quality, dry matter and cholesterol content of the egg. Kitchen‐extruded eggshell contained 98.52, 4.24, 29.75 and 14.82% DM, CP, Ca and P, whereas hatchery‐extruded eggshell contained 99.20, 13.80, 25.53 and 13.87% DM, CP, Ca and P, respectively. Limestone and Oyster shells contained 99.60 and 99.51% DM, and 37.12 and 35.20% Ca, respectively. Body weight, egg, hen day and egg mass production, and FCR did not differ among diets (*p* > .05). Egg production tended to increase with the increase of hen‐housed egg production (*p* < .001) in D_6_, followed by D_2_, D_5_, D_3_, D_4_ and D_1_, respectively. The lowest production cost and the highest net profit were observed in D_6_, followed by D_2_, D_4_, D_5_, D_1_ and D_3_, respectively. Diet with 8% Ca sources performed better than the diets with 4% Ca sources in terms of egg quality and dry matter content, where D_6_ was comparable to D_2_ or D_4_. The weight of egg, albumen, yolk, eggshell, dry yolk and yolk–albumen ratio increased, while dry albumen and eggshell weight, eggshell strength and thickness, Haugh unit, yolk index and egg‐specific gravity decreased with the increase in bird's age. The cholesterol content of yolk was statistically similar among diets. Therefore, no adverse effect of calcium sources on the production of laying hen was observed. Of these, extruded eggshell especially the 8% extruded eggshell may be beneficial to use in the diet of laying hen for producing a quality, safe and profitable egg.

## Graphical abstract

The extruded eggshell contains a large amount of Ca, P and a small amount of CP. The 8% extruded eggshell increased egg production, net profit and egg quality traits, but decreased FCR and production cost. The extruded (8%) eggshell may be beneficial to use in the diet of laying hen for producing a quality, safe and profitable egg.

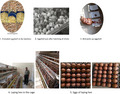

## 1 INTRODUCTION

Nowadays, there has been a steep rise in poultry production in the world which affects conventional feed ingredients leading to shortage and increase the cost of conventional feed ingredients. Hence, poultry scientists are trying to substitute conventional feed ingredients with cheaper unconventional feed resources to produce safe and cost‐effective poultry products. The feed account for about 60%–65% of the total production cost (Singh, 1990).

The eggshell contains 94% CaCO_3_, 1% calcium phosphate, 1% magnesium carbonate and 4% organic substances (Thapon & Bourgeois, 1994). Calcium carbonate from oyster shells contains lead vestige among the other potential toxic elements such as aluminium, cadmium and mercury. In this case, eggshell has a great advantage for not containing toxic elements.

Laying hens need Ca to lay and have strong eggshells. If hens do not get enough Ca in diet, their bodies pull Ca from their bones. As birds have no sweat gland, they rely on the panting system to dissipate excessive heat from the body. This causes excessive loss of carbon dioxide (CO_2_) which forms calcium carbonate (CaCO_3_) in the presence of Ca in the uterus. The net result is lower eggshell quality with soft or thin‐shelled eggs. This is why more calcium is needed to provide in layer ration. Eggshell is a good source of Ca, and the hens prefer this Ca very much even more than crust oyster shell or limestone. Large amounts of extruded eggshells in restaurants, hatchery, kitchen and egg product factories are thrown out as wastes every day. Extruded eggshell with membrane can be used as a source of Ca and a small amount of protein in layer diets without any adverse effects on egg production and egg quality (Gongruttananun, 2011; Liehovnikova, 2007). Sheideler (1998) has reported that extruded eggshell with a large particle size of limestone or oyster shell improved the eggshell quality. Therefore, the present study was aimed at determining the effect of extruded eggshell substituting limestone or oyster shell on egg production performance, egg and eggshell quality of laying hen for producing a quality, safe and cost‐effective egg.

## 2 MATERIALS AND METHODS

### 2.1 Collection and process of eggshell, limestone and oyster shell

Kitchen and hatchery‐extruded eggshell were collected from the student Hall of BSMRAU, restaurant and the hatchery, washed with fresh water and allowed to boil at a temperature of 100°C for 3–4 min, as a result all pathogenic bacteria, viruses and parasites were destroyed. In addition, the eggshells were dried under the sun, ground using a grinder and then stored. Limestone and oyster shells were collected from the local market.

### 2.2 Nutrient analysis of extruded eggshell, limestone and oyster shell

The nutrient content of kitchen and hatchery‐extruded eggshell, limestone or oyster shells were determined by a proximate analysis and spectrophotometric method (Ca & P) (AOAC, 2011) at the laboratory of the Department of Livestock Services, Dhaka, Bangladesh.

### 2.3 Feeding trial

A total of 120 ready‐to‐lay pullet, 18‐week‐old ISA Brown were collected from CP Bangladesh Company Ltd. and distributed into six dietary groups, namely D_1_ (diet with 4% limestone), D_2_ (Diet with 8% limestone), D_3_ (diet with 4% oyster shell), D_4_ (Diet with 8% oyster shell), D_5_ (Diet with 4% eggshell) and D_6_ (Diet with 8% eggshell) having 20 pullets in each dietary group. The birds were reared in an individual cage management system providing standard management practices as per standard given by the breeder during the experimental period (Table 1).

**TABLE 1:** Composition of diet used in the experiment

Ingredients	Diet (D) in Kg					
	D_1_	D_2_	D_3_	D_4_	D_5_	D_6_
Maize (Yellow corn)	61.00	63.00	61.50	60.00	64.00	62.50
Soybean meal	21.00	20.00	21.00	21.00	18.50	18.50
Rice polish	4.50	3.50	4.00	5.50	2.00	4.00
Protein concentrate	5.00	5.00	5.00	5.00	6.00	5.50
Limestone	4.00	8.00	—	—	—	—
Oyster shell	—	—	4.00	8.00	—	—
Eggshell	—	—	—	—	4.00	8.00
DCP	4.00	—	4.00	—	5.00	1.00
Salt	0.50	0.50	0.50	0.50	0.50	0.50
Total	100.00	100.00	100.00	100.00	100.00	100.00
Calculated Composition:						
Crude protein (%)	18.38	18.01	18.37	18.41	18.24	18.39
ME kcal/kg	2,775.94	2,787.89	2,777.04	2,773.74	2,769.94	2,768.14
Ca%	3.03	3.39	2.95	3.25	2.97	2.97
Available P%	1.24	0.51	1.23	0.53	1.94	1.82
Lysine	0.96	0.93	0.96	0.96	0.92	0.91
Methionine	0.29	0.28	0.29	0.29	0.28	0.28
Tryptophan	0.53	0.53	0.53	0.53	0.53	0.52

Note

Protein concentrate contains 62.30% CP, 8.10% crude fat, 3.24% phosphorus, 6.37% calcium, 19.30% ash, 8.36% moisture, 2,800 KcalME/kg, 1.78% methionine, 1.37% cystine, 3.87% lysine, 5.40% leucine, 1.91% isoleucine, 2.65% threonine, 3.68% valine and 0.53% tryptophan. However, in the present study only the CP, ME, Ca, P, lysine, methionine and tryptophan were calculated. Soybean meal contains 44% CP, 2,244 KcalME/kg, 0.25% Ca, 0.60% P, 2.85% lysine, 0.65% methionine and 0.60% tryptophan

+D_1_, Diet with 4% limestone; D_2_, Diet with 8% limestone; D_3_, Diet with 4% oyster shell; D_4_, Diet with 8% oyster shell; D_5_, Diet with 4% eggshell and D_6_, Diet with 8% eggshell; ME, Metabolizable energy, DCP, Di‐calcium phosphate.

### 2.4 Egg quality and dry matter content of egg

A total of 738 eggs from six dietary groups at 28, 36, 48, 56 and 68 weeks of age of the bird having 24–27 eggs/diet/age group were taken to determine egg quality traits. Ninety eggs from six dietary groups at 28, 36, 48, 56 and 68 weeks of age of the bird having three eggs/diet/age group were taken to determine dry matter content of the egg. A total of 21 eggs from six dietary groups and commercial farming eggs at 32, 44 and 56 weeks of age of the bird were taken to determine the cholesterol content of egg yolk.

### 2.5 Data recording

The nutrient content of eggshell, limestone and oyster shell was evaluated. Body weight, feed intake and egg and egg mass production were recorded fortnightly, mortality was recorded when occurred during the laying period. Hen day and hen‐housed egg production were calculated during the experimental period.

Egg quality and dry matter content of egg at 28, 36, 48, 56 and 68 weeks of age of the bird were recorded. Egg yolk cholesterol at 32, 44 and 56 weeks of age of the bird was recorded. FCR (Feed/dozen eggs and Feed/kg eggs), production cost and net profit (Tk/dozen eggs) were calculated during the experimental period.

### 2.6 Statistical analysis

The collected data were analysed using the Statistix10 computer package program (Statistix 10, 1985). Mortality of birds and cholesterol content of egg yolk were subjected to chi‐squared test and *t* test, respectively.

**Statistical model:** The following statistical model was used for the analysis of egg production performance data.

$$Y_{\text{ij}}=\;\mu \;+{\;D}_{i}\;+{\;e}_{\text{ij}}$$

where Y_ij_ is the observation of the *j*th number of individuals in the *i*th dietary group; µ is the overall mean; D_i_ is the fixed effect of the *i*th dietary group (*i* = 1, 2 ………6); and e_ij_ is the random error.

The following statistical model was used for the analysis of data of egg quality and dry matter content of the egg.

$$Y_{\text{ijk}}\;=\;\mu \;+{\;D}_{i}\;+{\;A}_{j}\;+\;{\left(D\;\times \;A\right)}_{\text{ij}}\;+{\;e}_{\text{ijk}}$$

where Y_ijk_ is the observation on the *k*th number of individuals in the *i*th dietary group and *j*th age group; µ is the overall mean; D_i_ is the fixed effect of the *i*th dietary group (i = 1, 2 ………6); A_j_ is the effect of the *j*th age group (j = 1, 2 ………5); and e_ijk_ is the random error.

## 3 RESULTS

### 3.1 Nutritive value of extruded eggshell, limestone and oyster shell

Kitchen and hatchery‐extruded eggshell contained 98.52% and 99.20% dry matter (DM), and 1.48% and 0.80% moisture, respectively. Limestone and Oyster shell contained 99.60% and 99.51% DM, and 0.40% and 0.49% moisture, respectively (Table 2). The value of crude protein (CP), Ca and available P were as 4.24, 29.75 and 14.82% in kitchen‐extruded eggshell, and 13.80, 25.53 and 13.87% in hatchery‐extruded eggshell, respectively. Limestone and oyster shells contained 37.12 and 35.20% Ca, respectively.

**TABLE 2:** Chemical composition of kitchen and hatchery‐extruded eggshell, limestone and oyster shell

Item	Dry matter (%)	Moisture (%)	CP%	Ca%	Av.P%
Kitchen‐extruded eggshell	98.52	1.48	4.24	29.75	14.82
Hatchery‐extruded eggshell	99.20	0.80	13.80	25.53	13.87
Limestone	99.60	0.40	—	37.12	—
Oyster shell	99.51	0.49	—	35.20	—

Abbreviations: Av. P, Available phosphorus; CP, Crude protein.

### 3.2 Egg production performance of laying hen‐fed diet with extruded eggshell, limestone and oyster shell for 365 days of laying period

Dietary groups were significantly different for feed intake, hen‐housed egg production, production cost (Tk/dozen or kg eggs) and net profit (*p* < .001), but not significantly different for body weight, egg production, hen day egg production, egg mass production, mortality and FCR (Feed/kg egg) (*p* > .05) (Table 3). Body weight of hen at the end of the experiment was almost similar among diets (*p* > .05). But the highest feed intake was observed in D_1_, followed by D_5_, D_6_, D_4_ and D_2_, respectively (*p* < .001). Evidently, but not significantly, the highest egg production, hen day egg production and egg mass production were observed in D_6_, followed by D_2_, D_5_, D_3_, D_4_ and D_1_, respectively. Feed conversion ratio (FCR) tended to decrease in D_6_, followed by D_2_, D_3_, D_5_, D_1_ and D_4_, respectively. The lowest production cost and the highest net profit were observed in D_6_, followed by D_2_, D_4_, D_5_, D_1_ and D_3_, respectively. Therefore, the diets with 8% Ca sources performed better than the diets with 4% Ca sources. Of these, D_6_ (8% eggshell) performed the best among the dietary groups in terms of egg production, production cost and net profit.

**TABLE 3:** Egg production performance of laying hen‐fed diet with extruded eggshell, limestone and oyster shell for 365 days of laying period

Traits	Diet (D)						LSD value and level of significance+
	D_1_	D_2_	D_3_	D_4_	D_5_	D_6_	
Body weight (g/bird)	2098.40	2028.50	2020.50	2070.20	2019.70	1965.60	119.070^NS^
Feed intake (g/bird)	43,244.00	42,456.00	42,895.00	42,609.00	43,182.00	43,073.00	377.680^***^
Egg production (No./bird)	290.76	310.40	299.37	295.61	302.55	314.58	18.250^NS^
Hen day egg production (%)	79.66	85.04	82.02	80.99	82.89	86.19	4.999^NS^
Hen‐housed egg production (%)	71.70	85.04	77.96	72.90	82.89	86.19	4.815^***^
Egg mass (g/bird)	17,454.00	18,186.00	17,908.00	16,872.00	17,534.00	18,937.00	1,490.400^NS^
Mortality (%)	10(2/20)	0	5(1/20)	10 (2/20)	0	0	ᵡ^2^=6.960^NS^
FCR (Feed/dozen egg)	1797.60	1657.60	1738.30	1747.00	1733.50	1648.90	106.930^NS^
FCR (Feed/kg egg)	2.52	2.36	2.44	2.59	2.51	2.29	0.213^NS^
Production cost (Tk/dozen egg)	80.61	73.07	81.28	75.82	79.26.22	69.61	3.321^***^
Production cost (Tk/kg egg)	103.46	93.97	102.58	100.91	104.00	87.36	6.551^***^
Net profit (Tk/dozen egg)	21.39	28.94	20.73	26.18	22.74	32.39	3.321^***^

Note

D_1_, Diet with 4% limestone; D_2_, Diet with 8% limestone; D_3_, Diet with 4% oyster shell; D_4_, Diet with 8% oyster shell; D_5_, Diet with 4% eggshell and D_6_, Diet with 8% eggshell; Price of egg (Tk/egg), 8.50; FCR, Feed conversion ratio.

^+NS^ p>0.05;

^***^ *p* <.001.

### 3.3 Egg quality traits of laying hen‐fed diet with extruded eggshell, limestone and oyster shell for 365 days of laying period

Egg quality traits differed significantly among dietary groups except for the traits, albumen and yolk width, yolk weight, membrane thickness and yolk–albumen ratio (*p* > .05) (Table 4). The highest egg weight was observed in D_1_, followed by D_3_, D_6_, D_4_, D_5_ and D_2_, respectively. The highest eggshell strength was measured in D_4_, followed by D_6_, D_2_, D_3_, D_5_ and D_1_, respectively. Therefore, the eggshell was stronger in diets with 8% Ca sources than in diets with 4% Ca sources. The diet D_6_ showed the highest yolk colour followed by D_5_, D_4_, D_2_, D_3_ and D_1_, respectively. Albumen and yolk width were almost similar among the dietary groups (*p* > .05). However, the highest albumen height was in D_1_, followed by D_2_, D_3_, D_5_, D_6_ and D4, respectively. The highest albumen weight was in D_3_ and D_1_, and the lowest in D_2_, D_4_, D_6_ and D_5_. Yolk weight was almost similar among dietary groups (*p* > .05), but the lowest eggshell with membrane weight was in D_3_ and D_5_, moderate in D_6_ and D_4_ and the highest in D_2_ and D_1_. The higher eggshell with membrane or eggshell thickness was observed in diets with 8% Ca sources compared to the diets with 4% Ca sources. Of these, the diet with 8% extruded eggshell was comparable to the diet with 8% limestone or oyster shell in terms of the eggshell thickness or eggshell strength. The highest Haugh unit was observed in D_2_, followed by D_1_, D_5_, D_3_, D_6_ and D_4_, respectively. The highest yolk index was measured in D_3_, D_6_, D_5_, D_2_ and the lowest in D_1_ and D_4_. The highest specific gravity was measured in D_2_, followed by D_4_, D_1_, D_6_, D_5_ and D_3_, respectively. The yolk–albumen ratio was almost similar among dietary groups (*p* > .05).

**TABLE 4:** Egg quality of laying hen‐fed diet with extruded eggshell, limestone and oyster shell for 365 days of laying period

Traits	Age (A)	Diet (D)							LSD value and level of significance+		
		D_1_	D_2_	D_3_	D_4_	D_5_	D_6_	Mean	D	A	D x A
Egg weight(g/egg)	A1	53.94	51.23	51.25	51.04	51.04	49.52	51.17	1.064^*^	0.946^***^	2.204^NS^
	A2	54.19	52.5	55.26	52.69	54.67	54.90	54.03			
	A3	56.80	55.30	54.79	55.35	54.26	54.57	55.18			
	A4	58.04	57.14	60.22	58.44	58.43	58.46	58.45			
	A5	60.11	59.24	60.43	59.67	57.96	60.22	59.60			
	Mean	56.41	55.08	56.39	55.44	55.27	55.52	55.69			
Eggshell Strength (Kg/egg)	A1	3.36	3.87	3.40	3.87	3.63	3.88	3.67	0.206^***^	0.183^***^	0.425^NS^
	A2	3.35	3.78	3.46	4.02	3.49	4.13	3.71			
	A3	3.29	3.57	3.51	3.87	3.21	3.62	3.51			
	A4	2.99	3.70	3.48	3.56	2.85	3.50	3.35			
	A5	3.55	3.59	3.66	3.83	3.41	3.72	3.63			
	Mean	3.31	3.70	3.50	3.83	3.32	3.77	3.57			
Yolk colour (DSM)	A1	8.29	8.88	8.75	9	9.04	8.25	8.7	0.259^***^	0.230^***^	0.535^***^
	A2	7.29	7.21	7.21	7.42	7.38	7.38	7.31			
	A3	7.21	7.50	7.50	7.54	7.63	7.33	7.45			
	A4	6.62	6.76	7.07	6.93	6.97	8.93	7.21			
	A5	6.58	6.83	6.54	6.38	6.88	6.92	6.69			
	Mean	7.20	7.44	7.41	7.45	7.58	7.76	7.47			
Albumen width (mm)	A1	78.95	74.58	77.76	77.43	76.61	78.38	77.28	1.183^NS^	1.051^***^	2.449^***^
	A2	75.72	74.65	76.03	75.28	75.81	75.21	75.45			
	A3	78.07	76.43	77.62	77.19	77.97	76.69	77.33			
	A4	76.42	76.04	78.63	77.34	79.43	78.24	77.68			
	A5	80.34	82.34	76.37	75.14	76.85	76.24	77.88			
	Mean	77.9	76.81	77.28	76.48	77.34	76.95	77.13			
Albumen height (mm)	A1	10.76	10.53	11.02	10.26	10.78	10.63	10.66	0.266^***^	0.236^***^	0.551^***^
	A2	10.96	10.58	10.00	9.32	9.89	9.74	10.08			
	A3	10.42	10.65	10.6	10.57	10.59	10.49	10.55			
	A4	10.05	10.25	10.24	10.18	10.13	10.43	10.21			
	A5	9.38	9.49	9.23	8.62	9.65	9.33	9.28			
	Mean	10.31	10.30	10.22	9.79	10.21	10.12	10.16			
Yolk width (mm)	A1	36.95	36.23	36.52	36.21	36.69	36.37	36.49	0.459^NS^	0.408^***^	0.951^NS^
	A2	37.42	36.75	37.58	37.21	37.05	37.11	37.19			
	A3	38.83	38.93	37.69	38.51	38.71	38.43	38.52			
	A4	38.57	38.09	39.39	38.73	39.09	38.90	38.79			
	A5	39.10	39.20	39.63	39.10	39.54	39.47	39.49			
	Mean	38.17	37.84	38.16	38.13	38.22	38.05	38.10			
Yolk height (mm)	A1	17.16	17.30	17.48	17.14	17.74	17.08	17.32	0.200^***^	0.178^***^	0.415^NS^
	A2	17.53	17.35	17.73	17.04	17.54	17.45	17.44			
	A3	17.75	18.00	18.01	17.93	17.70	18.10	17.92			
	A4	18.18	18.05	18.46	18.27	18.56	18.6	18.35			
	A5	18.65	18.83	19.31	18.78	19.10	19.30	18.10			
	Mean	17.86	17.91	18.20	17.83	18.13	18.11	18.01			
Albumen weight (%)	A1	33.83	32.85	32.72	32.12	32.42	31.48	32.57	0.754^***^	0.670^***^	1.562^NS^
	A2	34.66	32.88	34.88	32.58	34.39	34.26	33.94			
	A3	35.45	34.26	35.12	34.71	34.76	34.37	34.78			
	A4	36.22	35.16	37.75	36.39	36.73	36.17	36.40			
	A5	37.33	36.28	37.55	36.3	35.99	37.09	36.76			
	Mean	35.50	34.28	35.60	34.42	34.86	34.68	34.89			
Yolk weight (%)	A1	12.18	11.88	12.25	12.04	12.22	11.59	12.03	0.380^NS^	0.337^***^	0.786^NS^
	A2	13.04	13.16	13.40	12.96	13.19	13.59	13.22			
	A3	13.76	13.97	13.35	13.49	13.14	13.49	13.53			
	A4	14.50	14.44	15.18	14.77	14.61	14.61	14.69			
	A5	15.42	15.47	15.88	16.09	15.21	15.62	15.61			
	Mean	13.78	13.78	14.01	13.87	13.67	13.78	13.82			
Eggshell with membrane weight (%)	A1	6.31	6.56	5.88	6.39	6.05	5.89	6.18	0.168^***^	0.149^***^	0.347^NS^
	A2	6.22	6.27	5.99	6.22	5.96	6.24	6.15			
	A3	6.85	6.57	6.23	6.42	6.09	6.16	6.39			
	A4	7.07	7.35	7.06	7.07	6.90	7.41	7.14			
	A5	7.18	7.31	6.89	7.17	6.90	7.35	7.13			
	Mean	6.73	6.81	6.41	6.65	6.38	6.61	6.60			
Eggshell with membrane thickness (mm)	A1	0.477	0.427	0.433	0.437	0.423	0.406	0.434	0.008^***^	0.007^***^	0.017^***^
	A2	0.408	0.398	0.398	0.405	0.395	0.405	0.401			
	A3	0.417	0.406	0.407	0.412	0.388	0.405	0.406			
	A4	0.440	0.459	0.437	0.448	0.427	0.453	0.444			
	A5	0.400	0.431	0.418	0.416	0.395	0.432	0.415			
	Mean	0.428	0.424	0.419	0.424	0.405	0.420	0.420			
Eggshell thickness (mm)	A1	0.438	0.383	0.388	0.389	0.379	0.350	0.388	0.008^***^	0.007^***^	0.017^***^
	A2	0.373	0.367	0.361	0.37	0.357	0.370	0.366			
	A3	0.377	0.376	0.374	0.384	0.357	0.366	0.372			
	A4	0.402	0.422	0.401	0.417	0.393	0.421	0.401			
	A5	0.373	0.395	0.384	0.386	0.360	0.400	0.383			
	Mean	0.392	0.389	0.382	0.389	0.369	0.382	0.384			
Membrane thickness (mm)	A1	0.039	0.043	0.046	0.046	0.044	0.052	0.045	0.004^NS^	0.003^***^	0.007^*^
	A2	0.035	0.03	0.037	0.035	0.034	0.034	0.034			
	A3	0.041	0.03	0.032	0.029	0.031	0.038	0.033			
	A4	0.038	0.036	0.036	0.031	0.04	0.032	0.035			
	A5	0.027	0.035	0.035	0.03	0.035	0.032	0.032			
	Mean	0.036	0.035	0.037	0.034	0.037	0.038	0.036			
Haugh unit	A1	103.90	103.32	105.14	102.15	104.37	104.03	103.82	1.198^**^	1.064^***^	2.481^**^
	A2	104.50	103.12	100.12	97.75	99.87	99.12	100.75			
	A3	101.58	102.91	102.88	102.71	103.01	102.50	102.6			
	A4	99.86	100.96	100.34	100.34	100.19	101.46	100.53			
	A5	95.91	96.80	95.51	92.72	97.54	95.98	95.74			
	Mean	101.15	101.42	100.8	99.13	101	100.62	100.69			
Yolk Index	A1	0.465	0.479	0.479	0.474	0.484	0.470	0.475	0.007^*^	0.006^***^	0.015^NS^
	A2	0.469	0.473	0.472	0.458	0.474	0.471	0.470			
	A3	0.458	0.464	0.479	0.466	0.459	0.472	0.466			
	A4	0.473	0.475	0.47	0.473	0.477	0.479	0.474			
	A5	0.478	0.482	0.488	0.47	0.483	0.490	0.482			
	Mean	0.468	0.475	0.478	0.468	0.475	0.477	0.474	0.001^***^	0.001^***^	0.003^NS^
Specific gravity of egg	A1	1.098	1.103	1.095	1.101	1.098	1.098	1.099			
	A2	1.095	1.098	1.091	1.097	1.092	1.094	1.095			
	A3	1.098	1.098	1.094	1.096	1.094	1.094	1.096			
	A4	1.099	1.103	1.097	1.099	1.097	1.102	1.099			
	A5	1.098	1.100	1.095	1.099	1.098	1.099	1.098			
	Mean	1.098	1.100	1.094	1.099	1.096	1.097	1.097			
Yolk–Albumen Ratio	A1	0.361	0.365	0.376	0.375	0.378	0.389	0.371	0.011^NS^	0.010^***^	0.023^NS^
	A2	0.377	0.401	0.386	0.398	0.385	0.398	0.391			
	A3	0.391	0.409	0.382	0.390	0.380	0.395	0.391			
	A4	0.400	0.413	0.405	0.407	0.400	0.407	0.405			
	A5	0.413	0.429	0.425	0.444	0.423	0.423	0.426			
	Mean	0.388	0.403	0.395	0.403	0.392	0.398	0.397			

Note

D_1_, Diet with 4% limestone; D_2_, Diet with 8% limestone; D_3_, Diet with 4% oyster shell; D_4_, Diet with 8% oyster shell; D_5_, Diet with 4% eggshell and D_6_, Diet with 8% eggshell; A1, 28 weeks, A2, 36 weeks, A3, 48 weeks, A4, 56 weeks, A5, 68 weeks

^+NS^p>0.05;

^*^ *p* <.05;

^**^ *p* <.01;

^***^ *p* <.001.

Age affected the egg quality traits (*p* < .001). Egg weight, albumen and yolk width, yolk height, albumen weight, yolk weight, eggshell with membrane weight and yolk–albumen ratio increased with the increase in bird's age. However, eggshell strength, yolk colour, albumen height, eggshell with membrane thickness, eggshell thickness, membrane thickness, Haugh unit, yolk index and the specific gravity of egg decreased with the increase in bird's age.

Interaction between diet and age was observed for yolk colour, albumen width and height, eggshell with membrane thickness, eggshell thickness, membrane thickness and Haugh unit (*p* < .001) but no interaction of diet and age was found for egg weight, eggshell strength, yolk width, yolk height, albumen weight, yolk weight, eggshell with membrane weight, yolk index, specific gravity of egg and yolk–albumen ratio (*p* > .05).

### 3.4 Dry matter content of egg of laying hen‐fed diet with extruded eggshell, limestone and oyster shell for 365 days of laying period

There was no significant difference among dietary groups for dry matter content of egg of laying hen (*p* > .05) except the eggshell and dry eggshell weight and dry yolk weight (*p* < .05) (Table 5). The highest eggshell weight was observed in D_2_, followed by D_4_, D_1_, D_3_, D_6_ and D_5_, respectively. The diet D_2_ showed the highest dry yolk weight, followed by D_1_, D_4_, D_6_, D_5_ and D_3_, respectively. But the highest dry eggshell weight was observed in D_4_, followed by D_2_, D_6_, D_5_, D_3_ and D_1_, respectively. The other dry matter traits of egg were tended to increase in diets with 8% Ca sources than in diets with 4% Ca sources (*p* > .05). Thereof, the diet with 8% extruded eggshell was comparable to the diet with 8% limestone or 8% oyster shell in terms of the dry matter content of the egg.

**TABLE 5:** Dry matter content of egg of laying hen‐fed diet with extruded eggshell, limestone and oyster shell for 365 days of laying period

Traits	Age (A)	Diet (D)						Mean	LSD value and Level of Significance		
		D_1_	D_2_	D_3_	D_4_	D_5_	D_6_	Mean	D	A	D x A
Egg weight (g/egg)	A_1_	53.14	53.30	54.08	49.35	55.24	59.74	54.14	3.324^NS^	3.035^*^	7.433^NS^
	A_2_	59.05	55.08	61.03	51.60	55.15	60.59	57.08			
	A3	54.60	53.98	55.06	55.29	54.02	60.76	55.62			
	A4	54.24	58.74	61.31	59.27	58.98	53.80	57.72			
	A5	57.86	62.23	65.77	57.11	55.16	54.56	58.78			
	Mean	55.78	56.66	59.45	54.53	55.71	57.89	56.67			
Dry egg weight (%)	A_1_	30.21	30.09	29.83	31.15	30.70	30.31	30.38	0.967^NS^	0.883^NS^	2.163^NS^
	A_2_	31.90	30.86	30.08	29.93	28.64	29.91	30.22			
	A3	29.56	30.42	29.89	31.03	32.28	32.02	30.87			
	A4	30.20	31.27	29.41	31.52	30.38	30.35	30.52			
	A5	29.84	30.51	28.69	30.64	30.73	30.05	30.08			
	Mean	30.34	30.62	29.58	30.85	30.55	30.53	30.41			
Moisture (%)	A1	69.80	69.92	70.17	68.85	69.30	69.69	69.62	0.967^NS^	0.883^NS^	2.163^NS^
	A2	68.10	69.14	69.91	70.07	71.36	70.09	69.78			
	A3	70.44	69.58	70.11	68.97	67.72	67.98	69.13			
	A4	69.80	68.73	70.59	68.48	69.62	69.65	69.48			
	A5	70.16	69.49	71.30	69.36	71.27	69.95	69.92			
	Mean	69.66	69.37	70.42	69.14	69.45	69.47	69.59			
Albumen weight (%)	A_1_	61.58	61.85	64.00	62.07	65.30	62.11	62.82	1.531^NS^	1.397^NS^	3.423^NS^
	A_2_	60.18	61.94	62.26	62.51	63.55	62.64	62.18			
	A3	62.78	61.62	63.51	61.84	61.52	63.44	62.45			
	A4	60.18	59.58	63.44	60.18	62.45	61.08	61.19			
	A5	62.63	62.07	63.83	62.07	61.97	60.49	62.18			
	Mean	61.52	61.41	63.41	61.73	62.96	61.95	62.16			
Yolk weight (%)	A_1_	25.57	23.70	23.84	24.73	23.45	25.46	24.46	1.281^NS^	1.169^**^	2.864^NS^
	A_2_	25.25	25.11	25.49	25.24	24.88	26.24	25.37			
	A3	24.13	24.73	22.19	24.37	24.96	23.95	24.06			
	A4	27.02	27.67	23.25	27.61	25.64	26.19	26.23			
	A5	26.01	26.27	25.14	25.37	26.52	26.60	25.98			
	Mean	25.59	25.50	23.98	25.46	25.09	25.69	25.22			
Eggshell weight (%)	A_1_	11.84	14.39	11.71	12.90	10.80	11.53	12.19	0.795^*^	0.725^***^	1.777^***^
	A_2_	13.66	12.46	11.91	11.79	11.19	10.77	11.96			
	A3	13.02	14.77	13.91	13.59	13.20	12.34	13.47			
	A4	12.14	12.43	13.23	12.00	12.15	12.39	12.39			
	A5	10.80	10.15	9.91	11.28	10.60	12.16	10.82			
	Mean	12.29	12.84	12.13	12.31	11.59	11.84	12.17			
Dry albumen weight	A_1_	7.47	7.69	8.24	7.54	9.62	7.51	8.01	0.633^NS^	0.577^**^	1.415^NS^
(%)	A_2_	8.44	8.21	7.77	7.30	6.65	7.77	7.69			
	A3	7.89	7.32	8.26	7.99	8.55	9.05	8.17			
	A4	6.83	6.96	7.75	7.12	7.44	7.26	7.23			
	A5	7.34	7.21	6.89	7.13	7.61	7.29	7.25			
	Mean	7.59	7.48	7.78	7.41	7.98	7.78	7.67			
Dry yolk weight (%)	A_1_	14.04	13.24	12.18	12.74	12.11	12.95	12.88	0.785^*^	0.717*	1.756^NS^
	A_2_	13.95	13.26	13.10	13.32	12.79	13.48	13.32			
	A3	11.98	12.57	11.12	12.46	13.56	12.97	12.45			
	A4	13.48	14.12	11.61	14.55	12.93	13.21	13.32			
	A5	13.42	13.92	13.23	13.27	13.91	13.11	13.48			
	Mean	13.37	13.43	12.25	13.27	13.06	13.15	13.09			
Dry eggshell weight	A_1_	8.71	10.03	9.41	10.87	8.97	9.85	9.64	0.530^*^	0.484^***^	1.186^NS^
(%)	A_2_	9.51	9.39	9.22	9.31	9.20	8.66	9.21			
	A3	9.70	10.53	10.51	10.59	10.17	10.00	10.25			
	A4	9.89	10.19	10.04	9.86	10.02	9.87	9.98			
	A5	9.08	9.38	8.57	10.24	9.21	9.65	9.35			
	Mean	9.38	9.90	9.55	10.12	9.51	9.61	9.69			

Note

D_1_, Diet with 4% limestone; D_2_, Diet with 8% limestone; D_3_, Diet with 4% oyster shell; D_4_, Diet with 8% oyster shell; D_5_, Diet with 4% eggshell and D_6_, Diet with 8% eggshell; A1, 28 weeks, A2, 36 weeks, A3, 48 weeks, A4, 56 weeks, A5, 68 weeks

^+NS^ p > 0.05;

^*^ *p* < .05;

^**^ *p* < .01;

^***^ *p* < .001.

Age influenced the dry matter traits of the egg (*p* < .01) except the dry egg weight, moisture content and albumen weight (*p* > .05). Egg weight, yolk weight and dry yolk weight increased with the increase in bird's age, but the dry albumen, eggshell and dry eggshell weight decreased with the increase in bird's age. No interaction effect of diet x age was observed on dry matter content of egg (*p* > .05).

### 3.5 Cholesterol content of egg of laying hen‐fed diet with extruded eggshell, limestone and oyster shell for 365 days of laying period

No significant difference was observed among the dietary groups for the cholesterol content of egg yolk. Therefore, the cholesterol content of egg yolk was almost similar among the dietary groups (*p* > .05) (Table 6).

**TABLE 6:** Cholesterol content of egg of laying hen‐fed diet with extruded eggshell, limestone and oyster shell for 365 days of laying period

Diet (D)	Cholesterol (mg/100g)	LSD value and level of significance+
D_1_	275.85	104.310^NS^
D_2_	230.49	
D_3_	195.93	
D_4_	231.71	
D_5_	244.93	
D_6_	285.19	
D_7_	282.58	

Note

D_1_, Diet with 4% limestone; D_2_, Diet with 8% limestone; D_3_, Diet with 4% oyster shell; D_4_, Diet with 8% oyster shell; D_5_, Diet with 4% eggshell and D_6_, Diet with 8% eggshell.

^+NS^ *p* > 0.05.

## 4 DISCUSSION

### 4.1 Nutritive value of extruded eggshell, limestone and oyster shell

Extruded eggshell not only contains Ca and P but also contains crude protein (CP), because of the presence of eggshell membrane which contains crude protein. The estimated crude protein in the present study was higher than that reported by Lertchunhakiat et al. (2016). They found 2.14% CP in eggshell. However, the present study supported them for containing DM and Ca. They reported 99.40% DM and 29.87% Ca, a similar amount was measured in the present study. Hatchery‐extruded eggshell contained CP% which was higher than that of kitchen‐extruded eggshell. However, hatchery‐extruded eggshell contained Ca and P which was lower than kitchen‐extruded eggshell because of taking Ca and P by embryo from hatching egg during incubation. No previous work was found on the comparative nutritive evaluation of kitchen and hatchery‐extruded eggshell. On the other hand, limestone or oyster shell contained only Ca, which was higher than both the types of extruded eggshell. However, Olgun et al. (2015) reported a higher amount of Ca% in eggshells than in oyster shells. They showed 34.0, 32.3 and 32.0% Ca in limestone, eggshell and oyster shell, respectively. Yasothai and Kavithaa (2014) showed 33.5%–34.8% Ca in eggshell cited from Muir et al. (1976). Walton et al. (1973) showed 7.5%–8.1% crude protein in eggshell cited by Yasothai and Kavithaa (2014).

### 4.2 Egg production performance of laying hen‐fed diet with extruded eggshell, limestone and oyster shell for 365 days of laying period

Body weight, egg, hen day egg, egg mass egg production and FCR were almost similar among the dietary groups. Evidently, but not significantly, the highest egg and hen day egg production was observed in D_6_, followed by D_2_, D_5_, D_3_, D_4_ and D_1_, respectively. Similarly, FCR tended to be the lowest in D_6_, followed by D_2_, D_3_, D_5_, D_1_ and D_4_, respectively. Therefore, the present findings indicated that Ca sources had no adverse effect on egg production performances. The diet D_6_ showed the highest hen‐housed egg production, followed by D_2_, D_5_, D_3_, D_4_ and D_1_, respectively. These findings were supported by Gongruttananun (2011), Frontng, & Bergquist (2015). Olgun et al. (2015) partially supported these findings. They reported the significant effect of Ca sources on egg production, feed intake but not on body weight and FCR. Elsayed et al. (2014) reported that eggshell may be used as a Ca source in laying hen diet without any adverse effect on body weight, egg production and feed consumption. Saleh, Ahmed, et al. (2019) and Saleh, Kirrella, et al. (2019) reported the improved hen day egg production, egg weight, egg mass and FCR when they included the flaxseed and/ or fenugreek seed and cumin oil in the diet of laying hen that supports the present findings. The lowest production cost and the highest amount of net profit were observed in D_6_, followed by D_2_, D_4_, D_5_, D_1_ and D_3_, respectively. Thereof, it was found that the diets with 8% Ca sources performed better than the diets with 4% Ca sources in terms of egg production performances. However, the diet with 8% extruded eggshell (D_6_) performed the best in terms of egg production, production cost and net profit.

### 4.3 Egg quality traits of laying hen‐fed diet with extruded eggshell, limestone and oyster shell for 365 days of laying period

Albumen and yolk width, yolk weight, membrane thickness and yolk–albumen ratio were statistically similar among dietary groups but the other egg quality traits, namely egg weight, albumen weight, eggshell strength, eggshell weight and thickness, albumen and yolk height, yolk colour, Haugh unit, yolk index and specific gravity of egg differed significantly among dietary groups. For these traits, diets with 8% Ca sources performed better than the diets with 4% Ca sources. Of these, the diet D_6_ (8% extruded eggshell) was comparable to the diet with 8% limestone or 8% oyster shell in terms of egg quality traits. The present findings were consistent with the findings of Olgun et al. (2015). They found the increased egg and eggshell weight in the diet with extruded eggshell compared to the diet with oyster shells. Saleh, Ahmed, et al. (2019) and Saleh, Kirrella, et al. (2019) suggested to use flaxseed and/ or fenugreek seed and cumin oil in the diet of laying hen because of improving the weight of the egg, albumen, yolk and eggshell, eggshell thickness and yolk colour that also supports the present findings. The present findings contradicted the findings of Gongruttananun (2011) because they found no significant difference among the diets containing limestone or oyster shell for eggshell weight. Sheideler (1998) reported the improved eggshell quality in diet with the combination of eggshells and limestone or eggshells and oyster shells in comparison with the diet that included limestone or oyster shells only. Therefore, 8% extruded eggshell in the diet of laying hen might be beneficial to improve eggshell as well as egg quality.

As an effect of age, egg, albumen and eggshell weight, albumen and yolk width, yolk height and yolk–albumen ratio increased, while eggshell strength and thickness, yolk colour, albumen height, Haugh unit, yolk index and the specific gravity of egg decreased with the increase in the bird's age, which was supported by Mitrovic et al. (2010) and Tumova, and Ledvinka (2009). They reported an increased egg weight, albumen weight and yolk weight, and decreased Haugh unit, eggshell strength and thickness. Diet interacted with age for the egg quality traits except for egg weight, albumen width and weight, yolk width and weight, and eggshell strength.

### 4.4 Dry matter content of egg of laying hen‐fed diet with extruded eggshell, limestone and oyster shell for 365 days of laying period

No previous work on dry matter content of egg of laying hen influenced by Ca sources was found. Diets were significantly different for eggshell weight, dry eggshell weight and yolk weight. The other traits, namely egg and dry egg weight, albumen and dry albumen weight, yolk weight and dry yolk weight were statistically similar among dietary groups. However, the diets with 8% Ca sources performed better than the diets with 4% Ca sources in terms of the dry matter content of the egg. Of the 8% Ca sources, 8% extruded eggshell was comparable to the diet with 8% limestone or oyster shell.

As an effect of age, egg weight, yolk weight and dry yolk weight increased, while the eggshell weight, dry albumen weight and dry eggshell weight decreased with the increase in the bird's age. It indicates an increase in yolk size and decrease in moisture content of egg yolk with the increase in the bird's age. A lower eggshell weight was found, because of having the thinner eggshell with the increase in the bird's age. No interaction of diet x age was found for the dry matter traits except eggshell weight.

### 4.5 Cholesterol content of egg of laying hen‐fed diet with extruded eggshell, limestone and oyster shell for 365 days of laying period

No previous work was found on the cholesterol content of laying hen eggs that influenced by Ca sources. In the present study, no significant difference was observed among dietary groups for the cholesterol content of egg yolk of laying hen. Therefore, the cholesterol content of egg yolk was almost similar among dietary groups. Even the amount of cholesterol content of the commercial farming egg measured in the present study was also similar to the egg of test diets. However, Saleh, Ahmed, et al. (2019) and Saleh, Kirrella, et al. (2019) found the reduced cholesterol in blood plasma and egg yolk when they added flaxseed and/ or fenugreek seed and cumin oil in the diet of laying hen.

## 5 CONCLUSIONS

The present study reveals that kitchen‐extruded eggshell contains a higher amount of Ca and P but a lower amount of crude protein than the hatchery‐extruded eggshell. Limestone or oyster shell contains Ca only. The diet with 8% extruded eggshell performed the best among the dietary groups in terms of egg production, FCR and net profit. Egg quality traits and dry matter content of egg were better in diets with 8% Ca sources than in diets with 4% Ca sources. However, the diet with 8% extruded eggshell was comparable to the diet with 8% limestone or 8% oyster shell in terms of egg quality and dry matter content of the egg. The cholesterol content of the egg was almost similar among the dietary groups. Egg quality traits and dry matter content of egg except for egg, albumen, eggshell and yolk weight decreased with the increase in bird's age. Therefore, the extruded eggshell especially the 8% extruded eggshell may be beneficial to use in the diet of laying hen for producing a safe, quality and profitable egg.

## CONFLICT OF INTEREST

The authors declare that there is no conflict of interest regarding the publication of this article.

## AUTHOR CONTRIBUTION

**Mohammad Aminul Islam:** Conceptualization; Data curation; Formal analysis; Funding acquisition; Investigation; Methodology; Project administration; Resources; Software; Supervision; Validation; Visualization; Writing‐original draft; Writing‐review & editing. **Masahide Nishibori:** Conceptualization; Methodology; Writing‐review & editing.

## ETHICAL STATEMENT

The authors confirm that the ethical policies of the journal, as noted on the journal's author guidelines page, have been adhered to. Animal care and data collection procedures for the present study were approved by the Institutional Committee on Animal Care and Use in Research (ICACUR) of Bangabandhu Sheikh Mujibur Rahman Agricultural University (No. BSMRAU/DEAN/FVMAS/25/ICACUR/19). The authors confirm that they have followed EU standards for the protection of animals used for scientific purposes.

The experiments were carried out at Bangabandhu Sheikh Mujibur Rahman Agricultural University (BSMRAU), Gazipur −1706, Bangladesh.

### PEER REVIEW

The peer review history for this article is available at https://publons.com/publon/10.1002/vms3.544.

## References

[vms3544-bib-0001] AOAC . (2011). Official Methods of Analytical Chemists, 18th ed. Association of Official Analytical Chemists.

[vms3544-bib-0002] Elsayed, M. A., Basuony, H. A., & Hatab, M. H. (2014). The effect of calcium source in laying hen diet on egg and Tibia bone characteristics. Journal of Nuclear Technology in Applied Science, 2(4), 465–474.

[vms3544-bib-0003] Frontng, G. W., & Bergquist, D. (1990). Utilization of inedible eggshells and technical egg white using extrusion technology. Poultry Science, 69(11), 2051–2053. 10.3382/ps.0692051

[vms3544-bib-0004] Gongruttananun, N. (2011). Effects of eggshell calcium on productive performance, plasma calcium, none mineralization, and gonadal characteristics in laying hens. Poultry Science, 90, 524–529. 10.3382/ps.2010-01003 21248354

[vms3544-bib-0005] Lertchunhakiat, K., Saenphoom, P., Nopparatmaitree, M., & Chimthong, S. (2016). Effect of eggshell as a calcium source of breeder cock diet on semen quality. Agriculture and Agricultural Science Procedia, 11, 137–142. 10.1016/j.aaspro.2016.12.023

[vms3544-bib-0006] Lichovnikova, M. (2007). The effect of dietary calcium sources, concentration and particle size on calcium retention, eggshell quality and overall calcium requirement in laying hens. British Poultry Science, 48, 71–75. 10.1080/00071660601148203 17364543

[vms3544-bib-0007] Mitrovic, S., Pandurevic, T., Milic, V., Djekic, V., & Djrmanovic, V. (2010). Weight and egg quality correlation relationship on different age laying hens. Journal of Food Agriculture & Environment, 8, 580–583.

[vms3544-bib-0008] Muir, F. V., Harris, P. C., & Gerry, R. W. (1976). The comparative value of five calcium sources for laying hens. Poultry Science, 55, 1046–1051. 10.3382/ps.0551046

[vms3544-bib-0009] Olgun, O., Yidiz, A. O., & Cufadar, Y. (2015). The effect of eggshell and oyster shell supplemental as calcium sources on performance, eggshell quality and mineral excretion in laying hens. Indian Journal of Animal Research, 49(2), 205–209.

[vms3544-bib-0010] Saleh, A. A., Ahmed, E. A. M., & Ebeid, T. A. (2019). The impact of phytoestrogen source supplementation on reproductive performance, plasma profile, yolk fatty acids and antioxidative status in aged laying hens. Reproduction in Domestic Animals, 54(6), 846–854. 10.1111/rda.13432 30916364

[vms3544-bib-0011] Saleh, A. A., Kirrella, A. A., Dawood, M. A. O., & Ebeid, T. A. (2019). Effect of dietary inclusion of cumin seed oil on the performance, egg quality, immune response and ovarian development in laying hens under high ambient temperature. Animal Physiology and Animal Nutrition, 103(6), 1810–1817. 10.1111/jpn.13206 31518023

[vms3544-bib-0012] Sheideler, S. E. (1998). Eggshell calcium effects on egg quality and Ca digestibility in first‐ or third‐cycle laying hens. Journal of Applied Poultry Research, 7, 69–74. 10.1093/japr/7.1.69

[vms3544-bib-0013] Singh, R. A. (1990). Poultry Production, 3rd ed. Kalyani Publishers.

[vms3544-bib-0014] Statistix 10 . (1985). Analytical Software, 2105 Miller Landing Rd Tallahassee FL32312, USA.

[vms3544-bib-0015] Thapon, J. L., & Bourgeois, C. M. (1994). L’Oeuf et les ovoproduits. Lavousier Technique et Documentation, p. 344.

[vms3544-bib-0016] Tumova, E., & Ledvinda, Z. (2009). The effect of time of oviposition and age on egg weight, egg components weight and eggshell quality. Archiv Fur Geflügelkunde, 73(2), 110–115.

[vms3544-bib-0017] Vandepopuliere, J. M., Walton, H. V., & Cotterill, O. J. (1975). Nutritional evaluation of eggshell meal. Poultry Science, 54, 131–135. 10.3382/ps.0540131

[vms3544-bib-0018] Walton, H. V., Cotterill, O. J., & Vandepopuliere, J. M. (1973). Composition of eggshell waste from egg breaking plants. Poultry Science, 52, 1836–1841.

[vms3544-bib-0019] Yasothai, R., & Kavithaa, N. V. (2014). Chemical characterization of eggshell meal. International Journal of Science, Environment and Technology, 3(4), 1436–1439.

